# P-896. Long-term Impact of Automatic Stop Orders on Prolonged Broad Spectrum Antimicrobial therapy

**DOI:** 10.1093/ofid/ofaf695.1104

**Published:** 2026-01-11

**Authors:** nabaneeta dash, Geraldine Huynh, Kathryn E Timberlake, Michelle Science

**Affiliations:** The Hospital for Sick Children, Toronto, ON, Canada; The Hospital for Sick Children, Toronto, ON, Canada; The Hospital for Sick Children, Toronto, ON, Canada; The Hospital for Sick Children, Toronto, ON, Canada

## Abstract

**Background:**

Prolonged use of broad-spectrum antibiotics like meropenem and vancomycin is linked to development of antimicrobial resistance. Starting June 20, 2017, our hospital implemented a 72-hour stop date for vancomycin and meropenem orders in the electronic health system (EHS, Allscripts Sunrise Enterprise™ 2017-2018; Epic 2018-2024). This resulted in reduction in the number of prolonged vancomycin and meropenem courses ( >72 hours) from 44% to 38% (p=0.001). The objective of our study was to examine whether this reduction was sustained six years later by examining length of therapy (LOT) of vancomycin and meropenem courses.

**Figure:**
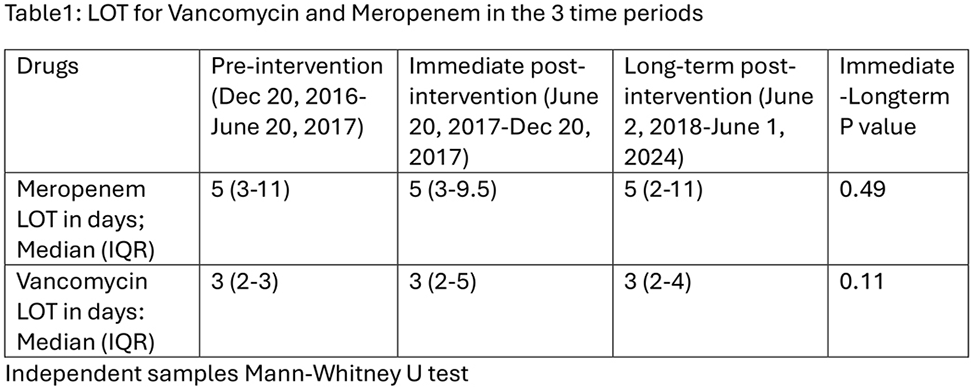

**Figure:**
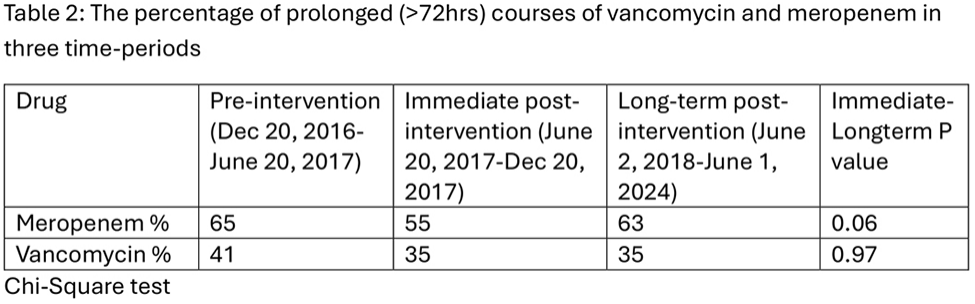

**Methods:**

All patients who received at least one dose of vancomycin/meropenem during the study period were included. LOT of an antibiotic was defined as the number of days a patient received at least one dose of the antibiotic. Courses interrupted for ≥24 hours were considered new courses. LOT data was compared across three periods: pre-intervention (Dec 20, 2016 – June 20, 2017), immediately post-intervention (June 20, 2017 – Dec 20, 2017), and long-term post-intervention (June 2, 2018 – June 1, 2024). Appropriateness of prolonged ( >72 hours) courses in the long-term post-intervention period were described (April 1, 2021, to March 31, 2023). Courses beyond 72 hours were considered appropriate if there was a resistant organism was isolated, or the course was approved by ID or ASP. The LOT was compared between periods using Chi-square.

**Figure 1:**
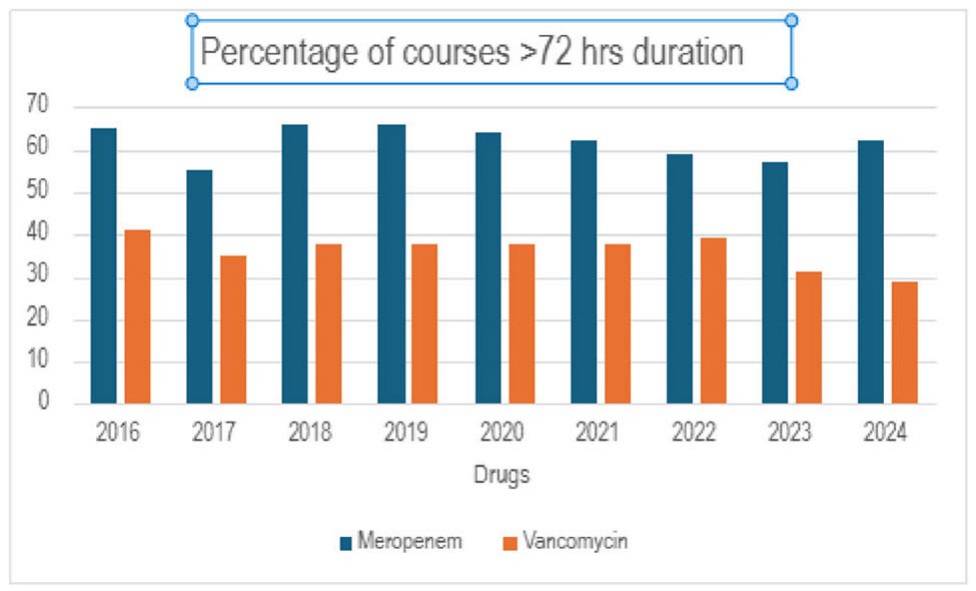
Percentage of Meropenem and Vancomycin courses >72 hrs from 2016-2024

**Results:**

There was also no statistical difference in the median LOTs for vancomycin or meropenem between the three time periods (Table 1). 63% of meropenem courses given between June 2, 2018 – June 1, 2024, were prolonged while 35% of vancomycin courses administered during same period was prolonged. During the period of assessment of appropriateness data 88% of vancomycin and 90% of meropenem courses were prescribed appropriately.

**Conclusion:**

Automatic stop orders can reduce the duration of antibiotic therapy and demonstrated 6-10% reduction in prolonged courses of vancomycin and meropenem immediately post-intervention. Unfortunately, this trend was not sustained, and further strategies are needed to sustain the impact of automatic stop dates.

**Disclosures:**

All Authors: No reported disclosures

